# Flux balance analysis of the ammonia-oxidizing bacterium *Nitrosomonas europaea* ATCC19718 unravels specific metabolic activities while degrading toxic compounds

**DOI:** 10.1371/journal.pcbi.1009828

**Published:** 2022-02-02

**Authors:** Gabriela Canto-Encalada, Diego Tec-Campos, Juan D. Tibocha-Bonilla, Karsten Zengler, Alejandro Zepeda, Cristal Zuñiga

**Affiliations:** 1 Facultad de Ingeniería Química, Universidad Autónoma de Yucatán, Mérida, México; 2 Department of Pediatrics, University of California, San Diego, California, United States of America; 3 Department of Bioengineering, University of California, San Diego, California, United States of America; 4 Center for Microbiome Innovation, University of California, San Diego, California, United States of America; CPERI, GREECE

## Abstract

The ammonia-oxidizing bacterium *Nitrosomonas europaea* has been widely recognized as an important player in the nitrogen cycle as well as one of the most abundant members in microbial communities for the treatment of industrial or sewage wastewater. Its natural metabolic versatility and extraordinary ability to degrade environmental pollutants (e.g., aromatic hydrocarbons such as benzene and toluene) enable it to thrive under various harsh environmental conditions. Constraint-based metabolic models constructed from genome sequences enable quantitative insight into the central and specialized metabolism within a target organism. These genome-scale models have been utilized to understand, optimize, and design new strategies for improved bioprocesses. Reduced modeling approaches have been used to elucidate *Nitrosomonas europaea* metabolism at a pathway level. However, genome-scale knowledge about the simultaneous oxidation of ammonia and pollutant metabolism of *N*. *europaea* remains limited. Here, we describe the reconstruction, manual curation, and validation of the genome-scale metabolic model for *N*. *europaea*, *i*GC535. This reconstruction is the most accurate metabolic model for a nitrifying organism to date, reaching an average prediction accuracy of over 90% under several growth conditions. The manually curated model can predict phenotypes under chemolithotrophic and chemolithoorganotrophic conditions while oxidating methane and wastewater pollutants. Calculated flux distributions under different trophic conditions show that several key pathways are affected by the type of carbon source available, including central carbon metabolism and energy production.

## Author summary

*Nitrosomonas europaea* catalyzes the first step of the nitrification process (ammonia to nitrite). It has been recognized as one of the most important members of microbial communities of wastewater treatment processes. Genome-scale models are powerful tools in process optimization since they can predict the organism’s behavior under different growth conditions. The final genome-scale model of *N. europaea* ATCC19718, *i*GC535, can predict growth and oxygen uptake rates with 90.52% accuracy under chemolithotrophic and chemolitoorganotrophic conditions. Moreover, *i*GC535 can predict the simultaneous oxidation of ammonia and wastewater pollutants, such as benzene, toluene, phenol and, chlorobenzene. *i*GC535 represents the most comprehensive knowledge-base for a nitrifying organism available to date. The genome-scale model reconstructed in this work brings us closer to understanding *N. europaea’s* role in a community with other nitrifying bacteria.

## 1. Introduction

Ammonia (NH_3_) as soluble ammonium (NH_4_^+^) is one of the main pollutants in industrial wastewater effluents, reaching concentration values from 5 to 1,000 ppm [1].

Biological nitrification is the primary method to remove NH_4_^+^ from wastewaters. This process involves the oxidation of NH_4_^+^ to nitrate (NO_3_^-^) via nitrite (NO_2_^-^). Nitrification is catalyzed by ammonia-oxidizing and nitrite-oxidizing bacteria in a two-step autotrophic process [2]. Several studies have used nitrifying consortia as mechanism to remove NH_4_^+^ and toxic pollutants (e.g., benzene, toluene, and phenol) simultaneously [3–6].

*Nitrosomonas europaea* (*Ne*) is a well-studied ammonia-oxidizing bacterium highly present in nitrifying consortia (usually from 1% to 5%) as well as an important microorganism in the nitrogen cycle [3,7]. *Ne* is typically grown with bicarbonate (HCO_3_^-^) as the major inorganic carbon substrate [8]. The HCO_3_^-^ assimilated is transformed to CO_2_ through the activity of the anhydrase carbonic enzyme [9]. The CO_2_ is further fixed by the Calvin-Benson-Bassham (CBB) cycle [10], producing energy by converting NH_3_ to hydroxylamine (NH_2_OH), and then to NO_2_^-^ (chemolithotrophic metabolism). Reactions catalyzed by the ammonia monooxygenase (AMO) and hydroxylamine oxidoreductase (HAO) [11]. AMO can metabolize various toxic pollutants present in wastewater, such as aromatic hydrocarbons and halogenated aliphatic compounds [12–15]. Physiological data highlighted have shown *Ne’s* versatility to utilize various substrates (pyruvate and fructose as sole organic carbon sources) under aerobic conditions [16]. However, the internal metabolic processes as the simultaneous NH_4_^+^ assimilation and toxic compounds oxidation, or the capability of an organic carbon mineralization (chemolithoorganotrophic metabolism) by *Ne* are not well characterized to date. Reduced modeling approaches have been used to elucidate *Ne* metabolism at a pathway level, resulting in two metabolic models: a metabolic network model with 51 reactions and a genome-scale metabolic model (M-model) with 1,102 total reactions [17,18]. Nevertheless, there are no M-models that enable elucidating flux distributions showing the mineralization of an organic carbon or the pollutant oxidation process under ammonia assimilation conditions. Here, we reconstructed a genome-scale model for *N*. *europaea* ATCC19718 using semi-automated methods [19,20]. The resulting model was manually curated to improve the quality of the phenotypic predictions. The model contains 1,149 reactions, and it is capable to accurately simulate growth under chemolithoorganotrophic and chemolithotrophic conditions while oxidating pollutants and methane.

## 2. Results

### 2.1 Reconstruction of the *Nitrosomonas europaea* metabolic network

We reconstructed an M-model of *N*. *europaea* ATCC19718 through a semi-automatic approach [19,20]. First, we reconstructed a draft model based on protein homology. We used the genome annotation of the proteome of *N*. *europaea* ATCC19718 from NCBI (RefSeq ID: NC_004757.1) [21] and three manually curated M-models as templates from the BiGG Database [22]. We selected *Escherichia coli* str. K-12 substr. MG1655 model, *i*ML1515 [23] as the first template, because *E*. *coli* is a gram-negative bacterium as *Ne*. Moreover, *i*ML1515 is the most complete genome-scale reconstruction of *E*. *coli* K-12 MG1655 to date. Then, as the second template, we selected the *Clostridium ljungdahlii* DSM 13528 model, *i*HN637 [24], since *C*. *ljungdahlii* is the only chemolithotrophic bacterium (such as *Ne*) present in the BiGG database. Finally, as the last template, the *Yersinia pestis* CO92 model, *i*PC815 [25], was chosen due to genome similarity (649 homologous metabolic genes) with *Ne* and the high quality of the model.

To generate the draft reconstruction (see Section 4.1) we used The RAVEN and COBRA Toolboxes [26,27] and to ensure the model connectivity and functionality (capability of the model to perform simulations) of the draft model, critical reactions were imported even though an equivalent hit in *Ne* proteome was not found. Draft reactions without a hit in *Ne* proteome were associated with genes from the templates, from now on referred to as exogenous genes. The generated draft model consisted of 1,056 metabolic reactions, 1,050 metabolites divided into three different compartments (cytoplasm, periplasm, and extracellular space) and, 734 genes, corresponding to 376 genes of *Ne* (identifier ALW85) and 358 exogenous genes.

Semi-automatic reconstruction ensures a functional model. However, during manual curation reactions are added or removed. For example, some CBB cycle reactions (1,5-biphosphate carboxylase and phosphoribulokinase), nitrification, methane oxidation, and pollutant oxidation metabolisms were added, while some reactions with exogenous genes were removed.

### 2.2 Refinement and gap-filling analysis

We used manual curation of the gene-protein-reaction (GPR) associations and gap-filling methods to improve the model properties (see Section 4.3). The original draft model consisted of 377 genes of *Ne* and 357 genes of the templates. After the manual curation, the number of exogenous genes decreased to 20, while *Ne* genes increased to 462. For more details of manual curation results, see S1 Text.

Gap-filling methods were carried out to find reactions specific for *Ne*. The nitrification and oxidative phosphorylation chain reactions were added based on previously described metabolic pathways [18,28,29]. Most of these reactions are not present in The BiGG Database (e.g., AMO, HAO, and reactions involving cytochrome c554, cytochrome c552, and membrane cytochrome c552). Moreover, none of the *Ne* models reconstructed so far included reactions related to methane and pollutant oxidation. We included complete oxidation pathways of methane, benzene, toluene, phenol and, chlorobenzene using the literature information [12–15,30]. A total of 82 gap-filled reactions categorized by growth condition are shown in S1 Table.

Reactions linking the CBB cycle with the pentose phosphate pathway (PPP) were also added, including the ribulose 1,5-bisphosphate carboxylase-oxygenase (RBPC). Overall, a total of 37 new reactions and 12 new metabolites were added. All these metabolites and reactions are shown in S2 and S3 Tables. For more details about the manual curation and gap-filling methods, see Section 4.3.2.

After the manual refinement process, all the exogenous genes were eliminated, and the *Ne* genes number increased to 535.

### 2.3 Model properties

**Fig 1:**
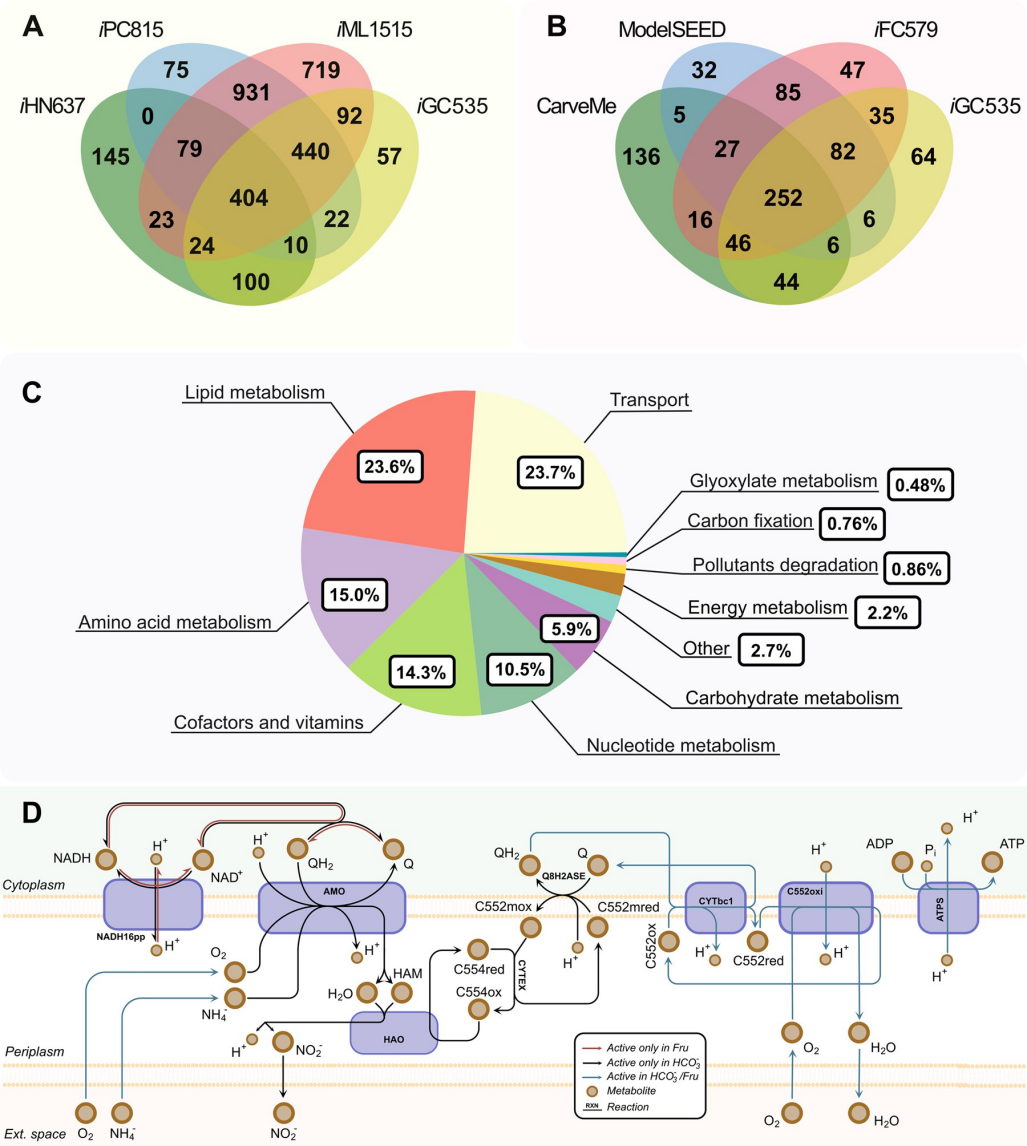
Features of *i*GC535. (A) Comparison of shared reactions among *i*GC535 and template models (*i*HN637, *i*PC815, *i*ML1515). (B) Comparison of shared genes among *i*GC535 and other *Ne* models. (C) Reactions distribution through the subsystems in the genome-scale model. (D) Electron transport chain simulated using fructose at high concentrations or HCO_3_^-^ as the sole carbon source. Abbreviations: NADH16pp, NADH dehydrogenase; AMO, ammonia monooxygenase; HAO, hydroxylamine oxidoreductase; CYTEX, cytochrome exchange; Q8H2ASE, ubiquinol synthase; CYTbc1, CytCbc1 reductase using ubiquinol-8; C552oxi, cytochrome c552 oxidase; ATPS, ATP synthase; QH_2_, ubiquinol; Q, ubiquinone; HAM, hydroxylamine; C554red, cytochrome c554 reduced; C554ox, cytochrome c554 oxidized; c552mox, membrane cytochrome c552 oxidized; c552mred, membrane cytochrome c552 reduced; C552ox, cytochrome c552 oxidized; C552red, cytochrome c552 reduced; Fru, fructose.

The final *Nitrosomonas europaea* ATCC19718 model (iGC535) consists of 1,114 metabolites, 1,149 reactions, and 535 genes (Table 1). In total, *i*GC535 shares 1,092 reactions with templates, of which 960 were obtained from the model *i*ML1515 (Fig 1A). Therefore, it was expected that *i*GC535 acquire most of its reactions from *i*ML1515. Both *E*. *coli* and *Ne* are gram-negative bacteria. Moreover, *i*ML1515 is the model with the highest genome coverage (1,515 genes) and amount of reactions (2,712).

**Table 1:** Comparative table of the properties of the genome-scale model of *Ne* (*i*GC535) and other models.

Model provenience	Reactions	Metabolites	Genes	Mass Imbalance metabolic reactions	Charge Imbalance metabolic reactions
ModelSEED	1,069	1,185	497	NA*	42
CarveMe	1,228	946	532	47	NA*
*i*FC578	1,102	1,014	578	NA*	129
Stoichiometric metabolic network	51	44	0	NA*	NA*
*i*GC535	1,149	1,114	535	0	0

*Not available

Model properties were compared with other genome-scale metabolic models of *Ne*. Fig 1B shows the breakdown of genes across different models. *i*GC535 has 471 genes in common with the other *Ne* models and 64 unique genes. *i*GC535 and *i*FC578 share the most amount of genes (415) since both have the highest genome coverage and were reconstructed semi-automatically (Fig 1B).

*i*GC535 was used to simulate inorganic carbon fixation (HCO_3_^-^), nitrite production, and organic carbon uptake (pyruvate and fructose). Moreover, *i*GC535 simulates the biotransformation of methane and wastewater pollutant (benzene, toluene, phenol, chlorobenzene) when ammonium and HCO_3_^-^ are the energy and carbon sources, respectively. These reactions and their genes were verified and curated manually through literature and information obtained from various databases, representing around 8.8% of the model’s total reactions. Most reactions (79%) are part of the transport, lipid, amino acid, nucleotide, cofactor, and vitamin biosynthesis (Fig 1C).

Table 1 shows a comparison among all the *Ne* models reconstructed to date. *i*GC535 shows an improvement in the amount of mass and charge-balanced reactions. In addition, it has been shown that *i*GC535 can simulate growth rates at lower ammonium uptake rates than the other *Ne* models (Table 2). Moreover, *i*GC535 is the only model that includes the oxidation pathways of methane and four pollutants.

**Table 2:** Comparative table of simulations performed under different growth conditions of the *Ne* genome-scale model (*i*GC535) and other models.

Condition	Constraint(s)	Evaluated Flux	Model	Simulation	Experimentally observed	
Chemolithoorganotrophy	Fructose uptake rate (0.0773 mmol/gDW/h)^a^	Growth rate (1/h)	ModelSEED	No growth		0.011
			CarveMe	No growth		
			*i*FC578	0.0113		
			*i*GC535	0.0106		
	Pyruvate uptake rate (0.0773mmol/gDW/h)	Growth rate (1/h)	ModelSEED	No growth		NA**
			CarveMe	No growth		
			*i*FC578	0.0057		
			*i*GC535	0.0053		
Chemolithotrophy	NO_2_^-^ production rate (23.61 mmol/gDW/h)^b^	Growth rate (1/h)	ModelSEED	No growth		0.062
			CarveMe	No growth		
			*i*FC578	0.015		
			*i*GC535	0.091		
	Ammonium uptake rate (2.09mmol/gDW/h)^c^	Oxygen uptake rate (mmol/gDW/h)	ModelSEED	0		3.07±0.084
			CarveMe	0		
			*i*FC578	0		
			*i*GC535	2.71		
	Ammonium uptake rate (1.266mmol/gDW/h)^c^	Oxygen uptake rate (mmol/gDW/h)	ModelSEED	0		2.03±0.05
			CarveMe	0		
			*i*FC578	0		
			*i*GC535	1.67		
Pollutants in culturing medium	Benzene uptake rate (1.258 mmol/gDW/h)^d^ Nitrite production rate (25.15mmol/gDW/h)^d^	Oxygen uptake rate (mmol/gDW/h)	ModelSEED	0		33.67
			CarveMe	0		
			*i*FC578	0		
			*i*GC535	33.58		
	Phenol uptake rate (1 mmol/gDW/h) Ammonium Uptake Rate (6 mmol/gDW/h)	Oxygen uptake rate (mmol/gDW/h)	ModelSEED	0		NA**
			CarveMe	0		
			*i*FC578	0		
			*i*GC535	0.0191		
	Toluene uptake rate (0.5206mmol/gDW/h) ^d^ Nitrite production rate (25.152mmol/gDW/h)^d^	Oxygen uptake rate (mmol/gDW/h)	ModelSEED	0		30.45±6.71
			CarveMe	0		
			*i*FC578	0		
			*i*GC535	33.44		
	Chlorobenzene uptake rate (1 mmol/gDW/h) Ammonium uptake rate (9 mmol/gDW/h)	Oxygen uptake rate (mmol/gDW/h)	ModelSEED	0		NA**
			CarveMe	0		
			*i*FC578	0		
			*i*GC535	12.1		
	Methane uptake rate (1.15 mmol/gDW/h)^e^ Ammonium uptake rate (1.74 mmol/gDW/h)^e^	Oxygen uptake rate (mmol/gDW/h)	ModelSEED	0		3.14±0.086
			CarveMe	0		
			*i*FC578	0		
			*i*GC535	3.02		
	Methane uptake rate (1.15 mmol/gDW/h) ^e^ Ammonium uptake rate (1.21 mmol/gDW/h) ^e^	Oxygen uptake rate (mmol/gDW/h)	ModelSEED	0		2.38±0.065
			CarveMe	0		
			*i*FC578	0		
			*i*GC535	2.356		

^a^ Hommes et al., 2003[16]

^b^ Sato et al., 1985 [31]

^c^ Hyman and Wood, 1983 [32]

^d^ Radniecki et al., 2008 [14]

^e^ Average methane uptake rate reported by Bédard and Knowles, 1989; Hyman and Wood, 1983; Jones and Morita, 1983 [32–34]

*Not available

Note: None of the conditions were simulated by the stoichiometric network

### 2.4 Model validation

The model was simulated and validated under chemolithotrophic and chemolithoorganotrophic growth conditions (Table 2). Fructose was used as organic carbon source under chemolithoorganitrophic conditions and HCO_3_^-^ under chemolithotrophic conditions. We also validated *i*GC535 using ammonium and HCO_3_^-^ as the energy and carbon sources, respectively, in the presence of methane and other pollutants. In all cases, ammonium was the inorganic nitrogen and energy source. Table 2 shows predicted phenotypes by *i*FC578 and the models of CarveMe and ModelSEED. Validation was performed using growth phenotypes experimentally observed under different uptake and secretion rates for each condition. Additionally, flux variability analysis was performed under each condition to obtain the feasible flux range of each metabolic reaction [35] (see Methods). S4 Table shows oxygen flux variability results (minimum and maximum) that were validated under chemolithotrophic conditions plus methane, toluene or benzene and the optimal value obtained while performing flux balance analysis.

#### 2.4.1 Chemolithoorganotrophic conditions

Under chemolithoorganotrophic conditions, when the fructose uptake rate and the nitrite production rate were 0.077 and 6.75 mmol/gDW/h, respectively, the experimentally observed growth rate of *Ne* was 0.011 1/h [16]. Model simulations using as constraints the fructose uptake and nitrite production fluxes reported by Hommes et al. [16] predicted 0.0106 1/h of growth rate, which has an absolute error of 4.0x10^-4^ mmol/gDW/h. This absolute error corresponds only to the 4.36% of error with the experimental data.

#### 2.4.2 Chemolithotrophic conditions

Under chemolithotrophic conditions using HCO_3_^-^ as carbon source and experimental nitrite production rate of 23.61 mmol/gDW/h, the experimentally observed growth rate of *Ne* was 0.062 1/h (Table 2) [31]. When this nitrite production rate was used as a constraint for *i*GC535 and *i*FC578 models, the predicted growth rates were 0.091 and 0.015 1/h, respectively. Furthermore, under these conditions Hyman and wood, 1983 [32] showed that *Ne* adjusts its ammonium uptake rate to 2.09 mmol/gDW/h and oxygen flux uptake to 3.07±0.084 mmol/gDW/h. Modeling predictions of *i*GC535 using this NH_4_^+^ uptake rate (2.09 mmol/gDW/h) as constraint, predicted an oxygen uptake rate of 2.71 mmol/gDW/h, while *i*FC578 predicted no growth (Table 2). Overall, the average accuracy of *i*GC535 predictions was 90.52%, remarkably higher than the average *i*FC578 accuracy (15.18%).

#### 2.4.3 Methane and pollutant oxidation conditions

Under chemolithotrophic conditions, methane and ammonium oxidation occurs simultaneously. Methane uptake is catalyzed by AMO, which requires oxygen to produce methanol that is eventually secreted to the medium. Experimentally, *Ne* is able to achieve maximum methane uptake rates between 0.34–1.96 mmol/gDW/h [32–34] when the ammonium uptake rate varies between 1.21 and 1.74 mmol/gDW/h [32]. Predicted flux distributions at a methane uptake rate of 1.15 mmol/gDW/h for the ammonium uptake rate of 1.21 mmol/gDW/h showed that the oxygen uptake flux was 2.356 mmol/gDW/h, which matches the experimentally observed oxygen uptake of 2.38±0.065 mmol/gDW/h [32]. Simulations at ammonium uptake rate of 1.74 mmol/gDW/h predicted 3.02 mmol/gDW/h oxygen flux uptake, diverging by 4% of the experimentally observed value of 3.14±0.086 mmol/gDW/h [30].

*Ne* can grow in the presence of toluene, using ammonium and HCO_3_^-^ as the energy and carbon sources [14]. AMO catalyzes the consecutive oxidation of toluene to benzyl alcohol and then to benzaldehyde as the final product [14]. Under these experimental conditions, the toluene uptake rate is 0.5206 mmol/gDW/h, the nitrite production rate 25.152 mmol/gDW/h and the oxygen uptake rate was 30.45±6.71mmol/gDW/h [14]. Optimal flux balance analysis simulations were highly accurate using as constraints the toluene uptake rate and the nitrite production rate since the model predicted an oxygen consumption rate of 33.44 mmol/gDW/h (Table 2). Additional experimental evidence under chemolithotrophic conditions [15] showed the capability of *Ne* to oxidize benzene to phenol by AMO activity under ammonium assimilation conditions. The phenol generated by this process is transported to the external medium. Radniecki et al. [14] observed an oxygen consumption rate of 33.67 mmol/gDW/h when the benzene uptake rate was 1.053±0.2059 mmol/gDW/h with a nitrite production rate of 25.15 mmol/gDW/h. *i*GC535 predicted an optimal oxygen uptake rate of 33.58 mmol/gDW/h when the benzene uptake rate was 1.258 mmol/gDW/h and a nitrite production rate of 25.15 mmol/gDW/h were set as constraints (Table 2). Overall, *i*GC535 accurately predicts growth and secretion phenotypes with over 90.52% accuracy.

### 2.5 Unraveling activation of specific metabolic capabilities using predicted flux distributions

#### 2.5.1 Co-activation of the pentose phosphate pathway, CBB cycle, and glycolysis

**Fig 2:**
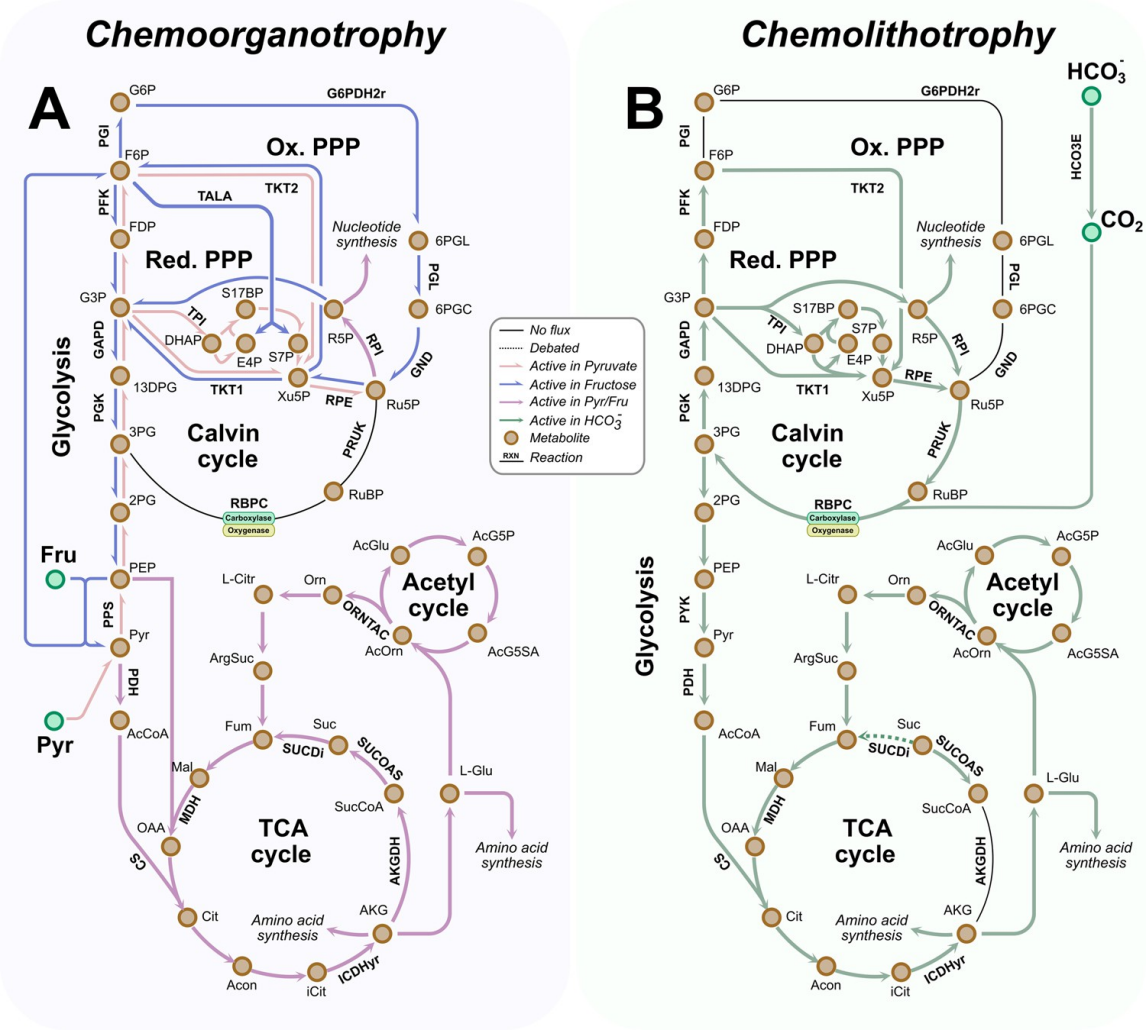
Map of the metabolic flux distributions predicted under chemolithoorganotrophic and chemolithotrophic conditions. The map shows the changes in the flux distributions under four different growth conditions. Ammonium is present under all conditions but changing the carbon source (fructose, pyruvate, and HCO_3_^-^). (A) Chemolithoorganotrophy metabolism. The fructose uptake rate was constrained to 0.746 mmol/gDW/h, and the ammonium uptake rate was 0.5mmol/gDW/h. (B) Chemolithotrophy metabolism. HCO_3_^-^ uptake rates were constrained to 4.35 mmol/gDW/h for high and 1.43 mmol/gDW/h for low. Abbreviations: HCO3E, carbonic anhydrase; PYK, pyruvate kinase; PPS, phosphoenolpyruvate synthasePGK, phosphoglycerate kinase; GAPD, glyceraldehyde 3-phosphate dehydrogenase; PFK, phosphofructokinase; PGI, glucose 6-phosphate isomerase; G6PDH2r, glucose 6-phosphate dehydrogenase; PGL, 6-phosphogluconolactonase; GND, phosphogluconate dehydrogenase; RPI, ribose-5-phosphate isomerase; RPE, ribulose 5-phosphate 3-epimerase; TKT, transketolase; TPI, triose-phosphate isomerase; PRUK, phosphoribulokinase; RBPC, ribulose 1,5-bisphosphate carboxylase-oxygenase; PDH, pyruvate dehydrogenase; CS, citrate synthase; MDH, malate dehydrogenase; SUCDi, succinate dehydrogenase; SUCOAS, succinyl-CoA synthetase; AKGDH, 2-oxoglutarate dehydrogenase; ICDHyr, isocitrate dehydrogenase; ORNTAC, ornithine transacetylase; G6P, D-Glucose 6-phosphate; F6P, D-Fructose 6-phosphate; FDP, D-Fructose 1,6-bisphosphate; G3P, Glyceraldehyde 3-phosphate; 13DPG, 3-Phospho-D-glyceroyl phosphate; 3PG, 3-Phospho-D-glycerate; 2PG, D-Glycerate 2-phosphate; PEP, Phosphoenolpyruvate; Pyr, Pyruvate; AcCoA, Acetyl-CoA; Fru, D-Fructose; Cit, citrate; Acon, Aconitate; iCit, Isocitrate; AKG, 2-Oxoglutarate; SucCoA, Succinyl-CoA; Suc, Succinate; Fum, Fumarate; Mal, L-Malate; OAA,Oxaloacetate; ArgSuc, L-Argininosuccinate; L-Citr, L-Citrulline; Orn, Ornithine; AcOrn, Acetylornithine; AcGlu, Acetyl-L-glutamate; AcG5P, Acetyl-L-glutamate 5-phosphate; AcG5SA, Acetyl-L-glutamate 5-semialdehyde; L-Glu, L-Glutamate; RuBP, D-Ribose 1,5-bisphosphate; Ru5P, D-Ribulose 5-phosphate; Xu5P, D-Xylulose 5-phosphate; DHAP, Dihydroxyacetone phosphate; E4P, Erythrose 4-phosphate dehydrogenase; S7P, Sedoheptulose 7-phosphate; S17BP, Sedoheptulose 1,7-bisphosphate; R5P, Ribose 5-phosphate; 6PGC, 6-Phospho-D-gluconate; 6PGL, 6-phospho-D-glucono-1,5-lactone.

*Ne* can grow using organic and inorganic carbon sources. To test changes in flux distributions, we used experimentally observed growth rates of 0.03 1/h [33] and 0.1 1/h [29] as constraints to determine HCO_3_^-^ uptake rates between 1.43 mmol/gDW/h and 4.35 mmol/gDW/h (Fig 2A).

Constraints for the organic carbon source fructose were set at high concentrations (0.746 mmol/gDW/h). Calculations regarding high fructose uptake rate are explained in section 2.6. The same consumption rate was established for pyruvate to ensure a high concentration of this metabolite.

When HCO_3_^-^ are present in the culture media, the CBB cycle enzymes are activated. Nevertheless, the CBB cycle is incomplete because of the lack of the enzyme NADPH-dependent glyceraldehyde 3-phosphate dehydrogenase (GAPD) [10]. This enzyme is replaced by the reversible glycolytic isoenzyme NADH-dependent GAPD in *Ne* metabolism [10] (Fig 2B). In other organisms, e.g., plants such as barley seedlings, spinach, pea leaves, etc., different genes encode for the glycolytic/CBB cycle enzyme phosphoglycerate kinase (PGK) [36–38]. But, *Ne* has only one form of PGK enzyme, which is used by the glycolytic pathway and the CCB cycle [9,29]. PGK is encoded by the gene ALW85_RS01720. The particularities of PGK and GAPD enzymes allow for the coupling of glycolysis and CBB cycle pathways, as shown in Fig 2.

In plants, the CBB cycle enzyme GAPD catalyzes the formation of glyceraldehyde-3-phosphate using NADPH as the electron donor [39]. However, *Ne* GAPD uses NAD/NADH as coenzymes rather than NADP/NADPH. The preference of GAPD towards NAD/NADH can be potentially associated with the differential activity of NAD and NADH in the respiratory chain (Fig 1D). Under heterotrophy, the NADH is oxidized in the electron transport chain by the NADH dehydrogenase (NADH16pp) enzyme to generate ATP. Nonetheless, when *Ne* is growing chemolithotrophically, NADH16pp catalyzes the reverse reaction, as shown in Fig 1D. Thus, NAD^+^ is reduced to NADH using ubiquinol as the electron donor. Model simulations predicted that NADH16pp and GAPD were the reactions with the highest production and consumption fluxes of NADH. This prediction suggest that the enzyme NADH16pp to produce the NADH needed by GAPD.

It has been shown that the CBB cycle is not activated under high fructose concentrations (Fig 2A). Fructose is transported into the cytosol by a phosphoenolpyruvate translocation group that forms pyruvate and fructose-6-phosphate (F6P). Then, pyruvate is used by the TCA cycle, while F6P is metabolized by glycolysis. Model simulations suggested that GAPD and NADH16pp fluxes are reversed when fructose is the carbon source. Thus, while GAPD is the largest consumer of NAD^+^, NADH16pp is the largest producer.

However, when pyruvate serves as the sole carbon source, GAPD produces NAD^+^ (Fig 2A). NAD^+^ production by GAPD occurs because part of the pyruvate flux that enters the organism goes to the gluconeogenesis pathway to produce G3P and F6P. These two metabolites are needed to synthesize ribulose 5-phosphate (Ru5P), a nucleotide precursor. Interestingly, NADH16pp also generates NAD^+^ but not NADH as during chemolithotrophic growth. A considerable amount of NAD^+^ is needed by the pyruvate dehydrogenase. This reaction connects the pyruvate with the TCA cycle.

PPP has an oxidative and reductive phase. The oxidative pathway (oPPP) regenerates NADPH (anabolic) that is used in the biosynthesis of the lipid. The reductive pathway (rPPP) produces glycolytic intermediaries (catabolic). *Ne* shares enzymes between the CBB cycle and the rPPP pathway, such as transketolase 1 (TKT1), ribulose 5-phosphate 3-epimerase (RPE), ribose-5-phosphate isomerase (RPI), transketolase 2 (TKT2), and transaldolase (TALA) (Fig 2). Under chemolithotrophy metabolism, the whole CBB/Glycolysis/rPPP superpathway was predicted to be active (except TALA) to synthesize F6P for D-ribose 1,5-biphosphate regeneration (Fig 2B). The predictions determined that the regeneration of NADPH occurs through NAD(P)^+^ transhydrogenase and not by oPPP. Since there is a high production rate of NADH, *Ne* uses this excess to synthesize NADPH.

When fructose is the carbon source, the Glycolysis/rPPP/oPPP superpathway is activated. The CBB cycle does not need to be activated since there is no presence of HCO_3_^-^ in the medium. The simulation predicted that oPPP is the greatest significant generator of NADPH. The Ru5P formed is used by rPPP to produce G3P, which is utilized by glycolysis. Nonetheless, when pyruvate is in the medium, some CBB cycle enzymes are activated to produce Ru5P (Fig 2A).

#### 2.5.2 TCA cycle

During chemolithotrophy growth, model simulations predicted that not all TCA cycle enzymes are activated (Fig 2B). Although *Ne* has the genes that encode for the complete TCA cycle, the 2-oxoglutarate dehydrogenase (AKGDH) enzyme is deactivated when HCO_3_^-^ is present. In other organisms such as aerobic heterotrophs, AKGDH catalyzes succinyl-CoA formation from 2-oxoglutarate and produces CO_2_ and NADH. In *Ne*, the NADH is supplied by NADH16pp. The 2-oxoglutarate is generated from the phosphoenolpyruvate and pyruvate formed by CBB/Glycolysis/rPPP superpathway. These two compounds are metabolized to oxaloacetate and acetyl-CoA, respectively, which are further condensed to citrate by the citrate synthase and the rest of the TCA cycle.

Unlike the chemolithotrophic growth, under chemolithoorganotrophic conditions, the simulations predicted the full activation of the TCA cycle (Fig 2A). By this route, some molecules of NADH and NADPH are produced.

Interestingly, *Ne* lacks malate synthase and isocitrate lyase. These two enzymes belong to the glyoxylate pathway. Plants and some bacteria use them to avoid the two decarboxylation steps catalyzed by AKGDH and isocitrate dehydrogenase [40]. *Ne* can avoid one decarboxylation step by AKGDH deactivation under chemolithotrophic conditions. Moreover, during chemolithoorganotrophic growth, *Ne* does not spare any of the decarboxylation steps. Thus, HCO_3_^-^ is excreted by the organism.

The succinate dehydrogenase (SUCDi) activity is unclear under chemolithotrophic conditions (Fig 2B). The simulation predicted a flux close to zero. The most significant amount of fumarate comes from the arginine biosynthesis pathway as a product of the argininosuccinate lyase reaction. Changes in this enzyme activity suggest that this pathway can potentially play an anaplerotic role depending on the growth conditions. For example, when simulations were performed using pyruvate or fructose as sole carbon sources, SUCDi was activated (Fig 2A). The simulations suggest that SUCDi activity changes because, unlike chemolithotrophic conditions, the succinyl-CoA synthetase is reversed, producing succinate. The only reaction that can consume the succinate is the SUCDi and thus avoid its accumulation.

#### 2.5.3 Electron transport chain

*Ne* obtains energy by ammonium assimilation. Fig 1C shows the flux distribution through the oxidative phosphorylation chain. NH_4_^+^ is oxidated to NO_2_^-^ by the activity of AMO and HAO. The metabolic flux continues through the electron transport chain to the terminal oxidase (C552oxi). The ATP synthase uses the proton gradient generated between the periplasm and the cytosol to produce ATP. Out of the total flux through HAO, 50% returns to AMO, 30.8% passes to the terminal oxidase, and 19.25% goes towards NADH16pp.

Unlike under chemolithotrophic conditions, model simulations performed at high fructose concentrations showed that the NADH16pp reduces an oxidized ubiquinol molecule using NADH as the electron donor (Fig 1D). The formed ubiquinol by NADH16pp and SUCDi (Figs 1D and 2A) reduces the cytochrome c552. Thus, the nitrite production decreases at 5.8x10^-4^ mmol/gDW/h, almost zero (S1B Fig).

#### 2.5.4 Methane and pollutant oxidation

*i*GC535 includes unique and complete pathways to oxidize methane and pollutants such as benzene, toluene, phenol, and chlorobenzene (~30 new reactions total). The most important enzyme in pollutant transformation is AMO, which catalyzes the oxidation of the contaminants and methane in the presence of ubiquinol. The oxygen required for oxidation depends on the methane or pollutant uptake rate. For example, if *Ne* uptakes ammonium at 1.266 mmol/gDW/h, the experimentally observed oxygen uptake rate is 2.03±0.05 mmol/gDW/h, as reported by Hyman and Wood, 1983 [32]. For the same ammonium uptake rate, *i*GC535 predicted an oxygen uptake flux of 1.67 mmol/gDW/h (Table 2). However, when the observed uptake rates of methane and ammonium were 1.15 and 1.2133 mmol/gDW/h, respectively, the oxygen uptake rate increases to 2.38±0.06 mmol/gDW/h [32]. Simulations predicted 2.356 mmol/gDW/h of oxygen uptake flux which matches with the experimentally observed value. Interestingly, even if more oxygen is used, the predicted growth rate decreases from 0.0037 1/h (without methane) to 5.56x10^-4^ 1/h (with methane). The predictions showed that during methane oxidation, more ubiquinol is needed. Ubiquinol is necessary for the oxidation of ammonium to hydroxylamine. Hydroxylamine participates in the electron transport through the oxidative phosphorylation chain and energy production. Therefore, by decreasing energy production, cell growth is reduced. Some studies have reported inhibition of ammonium oxidation by methane and aromatic compounds, triggering an energy drain [12,14,41,42].

All the products formed by pollutant oxidation are excreted to the external environment since *Ne* does not contain enzymes capable of mineralizing them [10,12,30,42,43]. End products of methane, benzene, phenol, toluene, and chlorophenol are methanol, phenol, hydroquinone, benzaldehyde, and chlorophenol, respectively, as it is reported by [14,15,30,41].

### 2.6 Flux distributions

**Fig 3:**
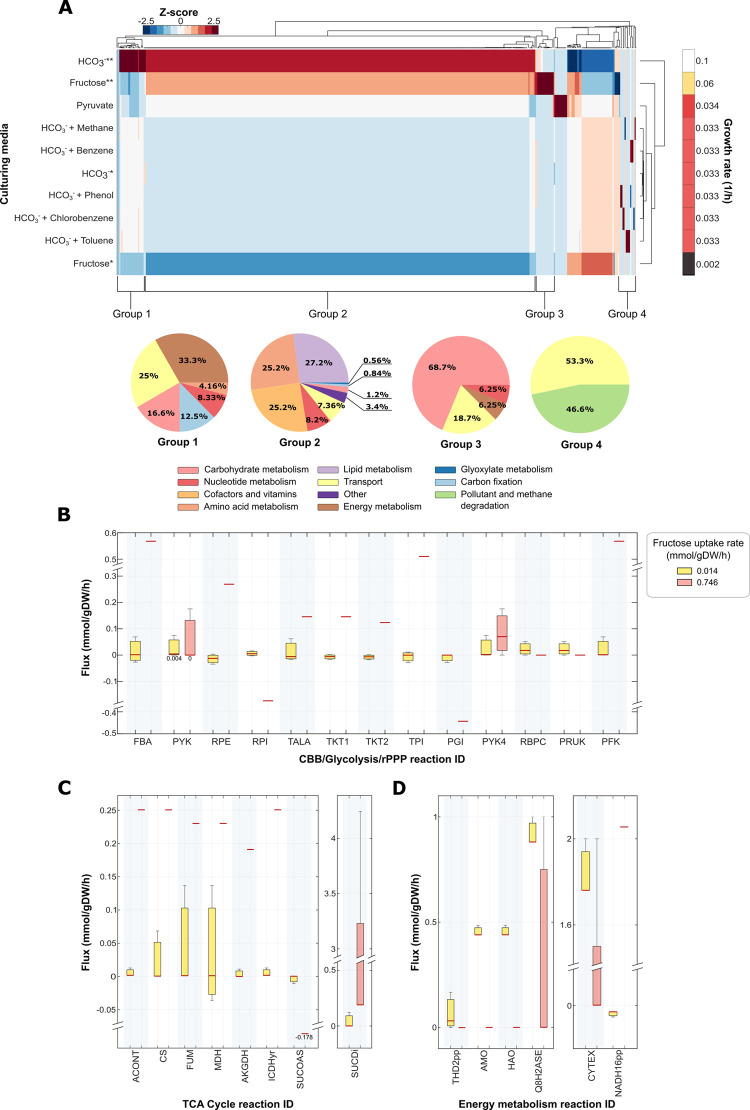
Flux distribution analysis under different growth conditions. (A) Hierarchical clustering of flux distributions under different carbon sources and growth conditions. The HCO_3_^-^ uptake rates were established at low and high levels, as we mentioned in Section 3.5.1. The pyruvate uptake flux (0.0773 mmol/gDW/h) used as a constraint in the simulations, resulted from the experimentally observed growth rate of 0.034 1/h [16] when *Ne* is grown using pyruvate as the organic carbon source. The methane and pollutant uptake rates were constrained to 1 mmol/gDW/h under the low HCO_3_^-^ level condition. We used standararized Z-scores to normalize predicted fluxes. Z-scores represent negative and positive values in a blue to red color scale. The X-axis shows the metabolic reactions, and the Y-axis shows different growth conditions. * means low uptake rates; ** means high uptake rates. Group 1: 24 reactions; Group 2: 353 reactions; Group 3: 16 reactions; Group 4: 15 reactions. (B) Change in flux predictions of CBB cycle, glycolysis, rPPP reactions when fructose uptake is at low or high concentration. (C) Change in flux predictions of TCA cycle reactions when fructose uptake is at low or high concentration. (D) Change in flux predictions of energy metabolism reactions when fructose uptake is at low or high concentration. Abbreviations: FBA, Fructose-bisphosphate aldolase; PYK, pyruvate kinase; RPE, ribulose 5-phosphate 3-epimerase; RPI, ribose-5-phosphate isomerase; TALA, transaldolase; TKT, transketolase; TPI, triose-phosphate isomerase; PGI, glucose 6-phosphate isomerase; RBPC, ribulose 1,5-bisphosphate carboxylase-oxygenase; PRUK, phosphoribulokinase; PFK, phosphofructokinase; ACONT, aconitate hydratase; CS, citrate synthase; FUM, fumarase; MDH, malate dehydrogenase; AKGDH, 2-oxoglutarate dehydrogenase; ICDHyr, isocitrate dehydrogenase; SUCDi, succinate dehydrogenase; SUCOAS, succinyl-CoA synthetase; THD2pp, NAD(P)^+^transhydrogenase; AMO, ammonia monooxygenase; HAO, hydroxylamine oxidoreductase; Q8H2ASE, ubiquinol synthase; CYTEX, cytochrome exchange; NADH16pp, NADH dehydrogenase.

Flux distributions were evaluated under different growth conditions (Fig 3). Fig 3A shows the hierarchical clustering of flux distributions ordered in descending order to the growth rate. Overall, higher fluxes are observed in simulations with higher growth rates. The increment in fluxes is due to the amount of energy yielded by the substrate, which in M-models that are in steady-state, the higher the energy production, the higher biomass production. Group 1 consisted of reactions related to carbon fixation and energy metabolism (Fig 3A).

Media with HCO_3_^-^ shows higher fluxes, which is in sync with the nitrification and carbon fixation processes occurring at greater rates under chemolithotroph conditions. Group 2 is the biggest one and contains the largest amount of reactions involved in lipid, amino acid, and vitamin metabolism. Simulations with greater growth rates show higher fluxes. 68.7% of group 3 (Fig 3A) belongs to the carbohydrate metabolism reactions. The growth under high fructose conditions shows the maximum flux in this group. Glycolytic enzymes are activated, and fructose is mineralized. Group 4 contains all the reactions involved in pollutant and methane oxidation.

We predicted two different slopes in the growth kinetics when simulating a medium of a continuous increase in fructose consumption at a specific ammonium uptake rate (S2 Fig). The difference in slope suggested metabolic changes between two conditions. Interestingly the model predicted that, when fructose was at low concentration (0.014 mmol/gDW/h), there was no CO_2_ production, and CCB cycle enzymes were activated (Figs S1 and 3B). The set of activated enzymes of TCA cycle fluxes, rPPP, and energy metabolism under low fructose was like those active during chemolithotrophic growth of *Ne* (Figs S2, 3B, 3C, 3D and 2B). However, as fructose concentration increased (0.746 mmol/gDW/h), the CBB cycle enzymes were deactivated, and CO_2_ production was predicted (Figs S1B and 3B). An overview of the flux distribution under high fructose uptake is shown in Fig 2A.

Furthermore, the flux balance analysis calculates a positive flux in NADH16pp (Fig 3D), which means NAD^+^ production, instead of NADH, contrary to what occurs at low fructose uptake rates. Besides, the fluxes of nitrification process enzymes (AMO, HAO, Q8H2ASE, CYTEX) decreases (Fig 3D). As a result, the nitrite production rate drops to practically zero (S1B Fig). We observed that when there is greater fructose availability, the model channels fluxes to the production of energy through the breakdown of the organic carbon source. Nevertheless, when the amount of organic carbon source drops, the flux balance analysis predicts that *Ne* recovers energy through chemolithotrophic metabolism.

## 3. Discussion

### 3.1 Model reconstruction and refinement

The model *i*GC535 was highly curated and successfully validated under various growth conditions, achieving the highest growth phenotype accuracy (Table 2) compared with all the *Ne* available models to date. Out of all the *Ne* available models (5 total), only *i*GC535 and *i*FC578 can simulate growth and nitrite production under chemolithotrophic conditions. But only *i*GC535 incorporates the phosphorylate oxidation chain while being fully mass and charge balanced, enabling the simulation of pollutant transformation pathways and growth under both chemolithoorganotrophic and chemolithotrophic conditions. To our knowledge, *i*GC535 is the first genome-scale model of *Ne* able to predict flux distributions simultaneous oxidation of pollutant and ammonium.

Predicted growth phenotypes and fluxes across the network of *i*GC535 were validated using experimental data (>90% accuracy). Higher prediction accuracies were calculated for chemolithoorganotrophic growth with fructose as the sole carbon source, for oxygen uptake rates under chemolithotrophic conditions, and during methane and pollutant oxidation. The model also provides advanced quantitative insights at the metabolic level about chlorobenzene and phenol oxidation. The manual curation performed to *i*GC535 resulted in a metabolic network of *Ne* with high certainty about reaction addition and cofactors usage. For example, using the glycolytic isoenzyme NADH-dependent GAPD instead of NADPH-dependent GAPD. Chain et al. [10] suggested that *Ne* saves a significant amount of energy by reducing 3-phosphoglycerate through the NADH-dependent GAPD. Our model simulations showed that *Ne* carries flux through this reaction (-2.9597 mmol/gDW/h), thus optimizing energy utilization.

### 3.2 Model-driven insights into *Ne* metabolism

Currently, the complete TCA cycle has been characterized in *Ne* [10]. Experimental studies have suggested that all TCA cycle enzymes are active when *Ne* is growing anaerobically (nitrite as the electron acceptor) while using organic carbon as the carbon source [44,45]. Moreover, it has been also shown that other chemolithotrophs can oxidize organic carbon sources to survive [46]. Model simulations predicted that NADH16pp oxidizes NADH to NAD^+^ (2.05 mmol/gDW/h) when fructose is present at high concentrations. This flux is reversed when HCO_3_^-^ is the carbon source (-10.19 mmol/gDW/h). Adessi and De Philippis, 2013 [47] suggested that NADH production instead of NAD^+^ is due to the excess of reduced ubiquinol. Model simulations showed that *Ne* maintains healthy levels of ubiquinol by activating the reaction NADH16pp.

Flux distributions also showed that under chemolithotrophic growth, all TCA cycle enzymes were active except for AKGDH. This prediction agrees with the study done by Beyer et al. and Hooper et al. [44,48], who observed that all TCA cycle enzymes were active except for AKGDH in *Ne*. However, SUCDi was active at a low flux (8.0939x10^-5^ mmol/gDW/h). Experimental observations revealed low activity or no significant expression amount of SUCDi under chemolithotrophy growth. Even more, some attempts to measure SUCDi activity by Deutch, 2013 [49] were unsuccessful. We believe that *i*GC535 will provide insights into the experimental design to better understand SUCDi activity in *Ne*.

*i*GC535 provides high resolution at metabolic and electron transfer levels. For example, model simulations showed that 50% of the electron flux from HAO returns to AMO under chemolithotrophic conditions, 30.8% passes to the terminal oxidase, and 19.25% goes towards NADH16pp. Wood 1986 [50], proposed that four electrons are removed from hydroxylamine oxidation by HAO, and two electrons return to AMO, 1.65 passes to the terminal oxidase, and the rest goes to NADH16pp. For this to happen, experimental evidence showed that out of all the electron flux from HAO, 50% goes to AMO, 41% to the terminal oxidase, and 8.7% to NADH16pp. Although the prediction of the flux proportion that returns to AMO coincided with that reported in the literature, the other two percentages diverged. However, the simulation correctly predicted a higher electron flux through AMO than C552oxi, which is subsequently higher than the electron flux through NADH16pp.

### 3.3 Metabolism change at low fructose concentrations

The model predicted the increase in RBPC activity when the fructose concentration drops. Thus, CO_2_ is fixed. Other organisms, such as *Pseudomonas oxalaticus* OX1, have also shown a progressive increase in RBPC activity and CO_2_ fixation when acetate concentration (organic carbon source) decreases in formate-limited culture [51].

Overall, *i*GC535 is a reliable systems biology tool that will be the base to understand and generate new hypothesis about *Ne* metabolism under a great variety of growth conditions.

## 4. Methods

### 4.1 Draft model generation

The proteome sequence of *N*. *europaea* ATCC19718 was obtained from The NCBI Reference Sequence database (Refseq code: NC_004757.1, total proteins: 2,507) [21]. Protein sequences were aligned to build the first draft model using bidirectional BLAST for protein homology (BLASTp criteria of ≥ 40% identity, e-value ≤ 1e^-30^, and query coverage ≥ 50%). The initial draft was reconstructed following semi-automatic reconstruction methods [19,20]. *i*ML1515 [23] was used as the first template model, *i*HN637 as the second template, and *i*PC815 [25] as the last template. [24]. We generated an initial version of the draft model from the protein homology between *Ne* and the first template. New reactions were added to the initial draft model using the remaining templates. The generated draft model also contained genes from the models used as references, which were later removed during the model refinement.

### 4.2 Biomass objective function

The biomass objective function (BOF) includes key metabolites part of biomass composition. The stoichiometric coefficient of each metabolite in this modeling reaction enforces an overall growth rate at a certain energy rate, here 46.66 mmol/gDW/h. The biomass objective function (BOF) and the growth-associate ATP maintenance value (46.66 mmolATP/gDW) of *i*HN637 (template) were imported to *i*GC535 because of the physiological similarity between both *C*. *ijungdahlii* and *Ne* (e.g., chemolithotrophs, grow under diverse environments). Then, the stoichiometric coefficients of amino acids in the BOF were updated using the abundance of the amino acid in the proteome of *Ne*, following the standard protocol for generating genome-scale metabolic reconstructions [52]. The breakdown of the amino acids was confirmed by *Ne* proteomic data under chemolithotrophic conditions [53]. Mineral compounds in the BOF (e.g., copper, iron, manganese) of the model were established according to *N*. *europaea* mineral requirements [54]. The final breakdown of biomass components as protein, nucleotide, and lipid content in the *Ne* biomass was 45.9, 12.8, 29.5%, respectively. This BOF generated was used as the objective function in all simulations executed in this paper. The model was also constrained by the non-growth-associated ATP maintenance (NGAM) (0.45 mmolATP/gDW), which was imported from the *i*HN637 model too. The NGAM is represented in the model as the reaction ATPM.

### 4.3 Model refinement and quality control and quality analysis (QC/QA)

Model refinement included two main stages: first, manual curation of the gene-protein-reaction associations of the model, including new metabolic reactions from The BiGG Database, and second, gap-filling that identified what metabolites were disconnected in the metabolic network and which reactions were missing by pathway.

#### 4.3.1 Manual curation

In the first step of manual curation, the reactions with exogenous proteins in the GPR associations were reviewed using different bioinformatic databases (KEGG, Biocyc, etc.). Exogenous proteins were replaced with homologous proteins of *N*. *europaea* for each reaction using the Enzyme Commission (EC) number as reference to identify the protein functionality. As the second step, these reactions without *N*. *europaea* proteins associations were checked through BLASTp. We determined sequence similarity among the proteins of these reactions comparing *N*. *europaea* proteome against the proteins assigned in the GPR associations for other organisms in the same reactions of the BiGG database. We identified proteins based on BLASTp criteria of > = 40% identity, e-value < = 1e^-30^, and query coverage > = 50%. The last step of manual curation was performed in order to validate the quality of the GPR associations [19]. The proteins for each reaction of the draft model were manually reviewed based on the type of metabolic reaction, protein function, and cell compartment. All the reactions and GPR associations validated were distributed in three different compartments (periplasm, cytoplasm, and extracellular compartment).

Afterward, remaining reactions with exogenous genes in the GPR associations of the model were identified and analyzed through Flux Balance Analysis (FBA) from the COBRA Toolbox [27]. The remaining reactions with no flux according to the FBA analysis and exogenous GPR associations were removed from the M-model.

A total of 687 false-positive GPR’s in the initial draft model were corrected. It was considered a false positive GPR if the reaction had at least one *Ne* missing gene or at least one erroneous annotated gene.

#### 4.3.2 Gap-filling

The gap-filling process was performed in two steps: first, gap-filling of the metabolic pathways already present in the manually curated draft model, and second, the addition of new metabolic pathways to the model from different bioinformatics databases (S1 Table).

Gap analysis was executed to identify which metabolites were disconnected in the metabolic network and which reactions were missing by pathway. Disconnected reactions were manually added using different bioinformatic databases (e.g., KEGG, Biocyc). From these analyses, gap-filling was employed to manually connect pathways through the data retrieved (pentose phosphate pathway, glycolysis, TCA cycle, etc.). Subsequently, the second round of gap-filling was performed to connect the metabolites from the medium conditions retrieved using literature information [15] through algorithms to identify the reactions involved in the wastewater pollutants oxidation, carbon, and nitrogen sources assimilation. A total of four wastewater pollutants (benzene, phenol, toluene, chlorobenzene), and methane were identified in ammonium assimilation conditions. Moreover, three carbon sources were used as substrates (HCO_3_^-^, fructose, pyruvate). The reactions added in the gap-filling process with no GPR associations were annotated as orphan reactions. Ultimately, reaction fluxes were validated using FBA to verify the predicted internal fluxes.

During the second step of gap-filling, new *N*. *europaea* central metabolism pathways and gene associations were added to the refined model using semi-automatic algorithms. The names of new reactions and metabolites were assigned according to BiGG and SimPheny databases [22,55]. Reactions and metabolites with no information in both databases were added to the model according to the EC Number information, other bioinformatics databases (KEGG, Biocyc, etc.), reactions and metabolites detailed information (charge, formula, reversibility, direction, etc.), and other metabolic models of *N*. *europaea* [17,18]. The connectivity of reactions was confirmed while performing FBA to predict biomass production. For reactions added in the second step of gap-filling were tested their connectivity by constraining uptake of those metabolites in the model and performing FBA simulations.

QC/QA included balance check-ups to ensure mass and charge balance of all reactions added to the model. Ultimately, the final model was tested for free ATP, NADH, and NADPH production without inputs.

### 4.4 Constraints and growth simulations

The culture medium composition under chemolithotrophic conditions retrieved from the literature [54] was used as the M-model constraints. The uptake and secretion rates under chemolithoorganothrophic, methane oxidation, and wastewater pollutant oxidation were collected using experimental data about growth phenotypes [12–15,30]. The constraints related to mineral medium composition and reactions with no flux value under both conditions (chemolithotrophic and chemolithoorganothrophic conditions) are summarized in S5 Table. Growth simulations were performed in the COBRA Toolbox for MATLAB [27] using the flux balance analysis procedure [56] and Gurobi Optimizer Version 5.6.3 solver (Gurobi Optimization Inc., Houston, Texas). Ammonium was used as the principal nitrogen and energy source. Meanwhile, HCO_3_^-^ was set as the principal carbon source. The M-model metabolic flux distributions were calculated under ten different growth conditions (HCO_3_^-^ at low and high uptake rates; fructose at low and high uptake rates; pyruvate; HCO_3_^-^+methane; HCO_3_^-^+chlorobenzene; HCO_3_^-^+benzene, HCO_3_^-^+phenol, and HCO_3_^-^+toluene, https://github.com/cristalzucsd/Nitrosomonaseuropaea). The predicted fluxes by reaction (or by column) were normalized by Z-score using the Statistics and Machine Learning Toolbox of MATLAB. For more Z-score normalization details, see S1 Text. Reactions fluxes were analyzed to determine the pathways’ participation according to the medium conditions, specifically, the pathways involved in the core (metabolism energy production, amino acid, nucleotide and lipid metabolism, etc.) and pollutants oxidation.

Ultimately, a robustness analysis was performed to determine the *Ne* metabolic capability to use fructose (from 0 mmol/gDW/h to 1 mmol/gDW/h) under various ammonium assimilation rates (from 0.5 mmol/gDW/h to 6.5 mmol/gDW/h). Predicted growth rates were compared with the experimental data.

In conclusion, our model *i*GC535 was successfully validated under a broad variety of conditions. It is a powerful tool to unravel the metabolism of *Ne*, and it will be a great tool to understand nitrification in the context of microbial communities to optimize the wastewater treatment and nitrogen cycle behavior. *i*GC535 represents the most comprehensive knowledge-base for a nitrifying organism available to date.

### 4.5 Flux Variability Analysis

Flux Variability Analysis [35] was used to quantify the maximum and minimum fluxes of the reactions in each evaluated condition (Section 2.4) and under low and high concentrations of fructose. The constraints used for these analyses are shown in S5 Table. This algorithm calculates the optimal growth rate and automatically sets it as constraint for the lower and upper bound of the biomass reaction. Then, for every reaction *i*, each ${\mathrm{ma}x}_{v_{i}}$ and ${min}_{v_{i}}$ is solved, where *v* represent *fluxes*. The analysis was designed to exclude the formation of the loops [57] by applying the linear loop-law constraints described by Schellenberger, et al., 2011 [58] to a COBRA mixed-integer linear programming (MILP) problem, which includes the fluxes of each internal reactions of the model. All additional inputs were set to default values. For example, the optimal percentage was 100%, enabling to achieve 100% of the biomass objective function.

## Supporting information

S1 Text Methods and results description. (DOCX)

S1 Fig Flux prediction change under high and low fructose concentrations. (A) Flux prediction change of CBB cycle, glycolysis, rPPP reactions when fructose uptake is 0.014 (low concentration) or 0.746 (high concentration) mmol/gDW/h (ammonium uptake rate setting on 0.5 mmol/gDW/h). (B) Flux prediction change of exchange reactions when fructose uptake is 0.014 or 0.746 mmol/gDW/h (ammonium uptake rate setting on 0.5 mmol/gDW/h). (C) Flux prediction change of nucleotide metabolism reactions when fructose uptake is 0.014 or 0.746 mmol/gDW/h (ammonium uptake rate setting on 0.5 mmol/gDW/h. (D) Flux prediction change of oPPP reactions when fructose uptake is 0.014 or 0.746 mmol/gDW/h (ammonium uptake rate setting on 0.5 mmol/gDW/h. Abbreviations: FBA, Fructose-bisphosphate aldolase; GAPD, glyceraldehyde 3-phosphate dehydrogenase; PGK, phosphoglycerate kinase; PYK, pyruvate kinase; RPE, ribulose 5-phosphate 3-epimerase; RPI, ribose-5-phosphate isomerase; TALA, transaldolase; TKT, transketolase; TPI, triose-phosphate isomerase; PGI, glucose 6-phosphate isomerase; RBPC, ribulose 1,5-bisphosphate carboxylase-oxygenase; PRUK, phosphoribulokinase; PFK, phosphofructokinase; EX_co2_e, exchange reaction of CO2; EX_no2_e, exchange reaction of NO2; ADK, adenylate kinase; GND, phosphogluconate dehydrogenase; G6PDH2r, glucose 6-phosphate dehydrogenase; PGL, 6-phosphogluconolactonase.
(TIF)

S2 Fig Growth rates predicted by *i*GC535 under different uptake fluxes of ammonium and fructose. (TIF)

S3 Fig Color scale of the reaction fluxes Z-score normalized. (TIF)

S1 Table Gap-filled reactions by growth condition. (XLSX)

S2 Table New metabolites added to *i*GC535. (XLSX)

S3 Table New reactions added to *i*GC535. (XLSX)

S4 Table Maximum, minimum, and optimal solutions of iGC535 under different growth conditions. (XLSX)

S5 Table *i*GC535 Culture medium constraints for all conditions. (XLSX)

S6 Table Normalized fluxes by reaction. (XLSX)
