# Supplementary material for: Flux balance analysis of the ammonia-oxidizing bacterium Nitrosomonas europaea ATCC19718 unravels specific metabolic activities while degrading toxic compounds
Source: PLoS Comput Biol. 2022 Feb 2;18(2):e1009828. doi: 10.1371/journal.pcbi.1009828 (PMC8853641; doi:10.1371/journal.pcbi.1009828)
Supplement: S1 Text — (DOCX) [file pcbi.1009828.s001.docx]

Supplemental material

Flux balance analysis of the ammonia-oxidizing bacterium *Nitrosomonas europaea* ATCC19718 unravels specific metabolic activities while degrading toxic compounds

Short title: Genome-scale metabolic modeling of *Nitrosomonas europaea*

Gabriela Canto-Encalada^a^, Diego Tec-Campos^a,b^, Juan D. Tibocha-Bonilla^b^, Karsten Zengler^b,c,d^, Alejandro Zepeda^a^, Cristal Zuñiga^b,^*

1. *Results*

2.1 Draft model generation

A draft model was generated using the proteome of *Ne*. The resulting draft model had 1,056 metabolic reactions and 1,050 metabolites divided into three different compartments (cytoplasm, periplasm, and extracellular space). The draft model imported the composition of the biomass objective function (BOF) of the first template, *i*HN637, a chemolithotrophic organism. Mineral compounds in the BOF (e.g. copper, iron, manganese) of the model were established according to *N. europaea* mineral requirements [1]. The final breakdown of biomass components as protein, nucleotide, and lipid content in the *Ne* biomass were 45.9, 12.8, 29.5%, respectively. The ATP maintenance was obtained from the *i*HN637 model (46.66 mmol/gDW). This BOF generated was used as the objective function in all simulations executed in this paper.

2.2 Manual curation

The draft model was further curated. New GPR associations were verified using multiple databases (e.g., BLASTp, KEGG, Biocyc, BRENDA, and MetaNetX) as well as available information from the literature. Reactions with genes from the templates (exogenous) were searched in KEGG to replace them with *Ne* genes (ALW85). After this step, the model contained 388 ALW85 genes and 346 genes from templates. In a second step, the remaining reactions with exogenous genes were retrieved from different organisms present in The BiGG database. Their protein sequences were aligned with protein sequences of *Ne* using bidirectional BLAST*.* Genes whose sequences passed the BLASTp parameters (see Methods) were assigned in the GPR associations. After this step, the ALW85 genes increased to 391 and the number of template genes decreased to 138.

The last step of refinement consisted of manual verification of all GPR associations one by one (see Methods). Reactions associated with endogenous genes that could not be verified went through a refinement process described for exogenous genes in step 2. After that, the total number of genes was 483, of which 462 were ALW85 genes and 20 template genes.

A total of 687 false-positive GPR's in the initial draft model were corrected. It was considered a false positive GPR if the reaction had at least one *Ne* missing gene or at least one erroneous annotated gene. The false-positive rate of the GPR association was determined by the genes into each *Ne* model reconstructed so far. We found that the false-positive rate was 13.8% for *i*FC579; 11% for ModelSEED; and 30% for CarveMe. Direct comparison by reactions was not possible since models use different naming.

2.5 Unraveling activation of specific metabolic capabilities using predicted flux distributions

2.5.1 Arginine biosynthesis

Unlike some bacteria, plants, and fungi [2,3], *Ne* lacks acetylornithine deacetylase in the arginine biosynthesis pathway. *i*GC535 simulations showed that under chemolithotrophic and chemolithoorganotrophic conditions, the acetylglutamate synthase reaction is activated, triggering the recycling of the acetyl group through the ornithine transacetylase (ORNTAC). ORNTAC transfers the acetyl group to N-acetyl glutamate from the N-acetyl ornithine, producing the ornithine necessary for arginine synthesis (Fig 2).

2.6 Methods

2.6.1 Z-score normalization

The Z-score normalization transforms the data to a new scale where the mean of the raw data is 0. Thus, the new normalized data indicates how many standard deviations they are from the real mean. For example, if a flux is normalized to 2.44, it means that the raw score is 2.44 standard deviations away from the mean. Normalized fluxes from negative to positive are present on a blue to red scale (S3 Fig). Then, the color in the figure will be deep red (S6 Table). If the original flux is below the mean, it will get a blue color, while if it is above the mean, the color will be red. Therefore, even if there is a very close flux value between conditions, the further it is from the mean, the more different the color. For example, in S6 Table, we show the RBPC reaction fluxes in the different growth conditions. As the reviewer said, the flux of RBPC reaction under high HCO3 concentrations condition (5.26 mmol/gDW/h) is three times higher than at low HCO_3_^-^ concentration (1.736 mmol/gDW/h). However, when the fluxes of RBPC reaction under all conditions are Z-score normalized, the value at low HCO3 concentrations is 0.09 obtaining a white color, while at high HCO_3_^-^ concentrations is 2.44 obtaining a deep red color.

Another example is the reaction PSSA160 (S6 Table). At high HCO_3_^-^ concentrations, the flux is 0.0109, while at low HCO_3_^-^ concentrations is 0.0036, three times lower. The mean of PSSA160 fluxes set is 0.0043, which indicates that the flux at high HCO_3_^-^ concentrations is above the mean, while at low concentrations is below the mean. Therefore, the normalized fluxes resulted in 2.3158 (red) and -0.2708 (blue), respectively.

References

1. Brenner DJ, Krieg NR, Staley JT, Garrity GM, editors. Bergey’s manual® of systematic bacteriology: volume two: The proteobacteria, part A introductory essays. 2nd ed. Lansing: Michigan State University Springer-Verlag; 2005. 146 p.

2. Sakanyan V, Petrosyan P, Lecocq M, Boyen A, Legrain C, Demarez M, et al. Genes and enzymes of the acetyl cycle of arginine biosynthesis in Corynebacterium glutamicum: Enzyme evolution in the early steps of the arginine pathway. Microbiology. 1996;142(1):99–108.

3. Slocum RD. Genes, enzymes and regulation of arginine biosynthesis in plants. Plant Physiol Biochem. 2005;43(8):729–45.
