# Supplementary figures and images for: Flux balance analysis of the ammonia-oxidizing bacterium Nitrosomonas europaea ATCC19718 unravels specific metabolic activities while degrading toxic compounds
Source: PLoS Comput Biol. 2022 Feb 2;18(2):e1009828. doi: 10.1371/journal.pcbi.1009828 (PMC8853641; doi:10.1371/journal.pcbi.1009828)

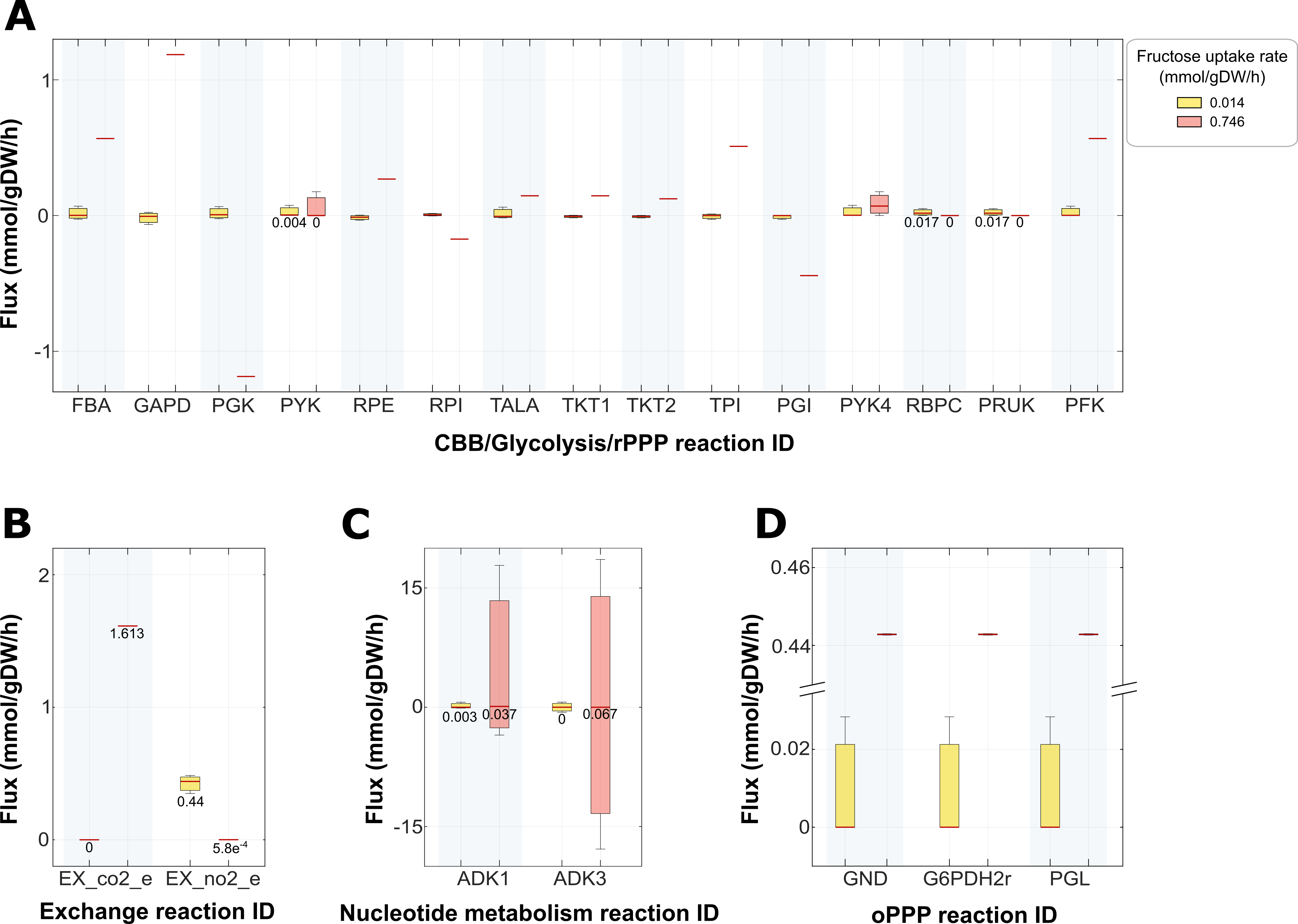

Supplement: S1 Fig — (A) Flux prediction change of CBB cycle, glycolysis, rPPP reactions when fructose uptake is 0.014 (low concentration) or 0.746 (high concentration) mmol/gDW/h (ammonium uptake rate setting on 0.5 mmol/gDW/h). (B) Flux prediction change of exchange reactions when fructose uptake is 0.014 or 0.746 mmol/gDW/h (ammonium uptake rate setting on 0.5 mmol/gDW/h). (C) Flux prediction change of nucleotide metabolism reactions when fructose uptake is 0.014 or 0.746 mmol/gDW/h (ammonium uptake rate setting on 0.5 mmol/gDW/h. (D) Flux prediction change of oPPP reactions when fructose uptake is 0.014 or 0.746 mmol/gDW/h (ammonium uptake rate setting on 0.5 mmol/gDW/h. Abbreviations: FBA, Fructose-bisphosphate aldolase; GAPD, glyceraldehyde 3-phosphate dehydrogenase; PGK, phosphoglycerate kinase; PYK, pyruvate kinase; RPE, ribulose 5-phosphate 3-epimerase; RPI, ribose-5-phosphate isomerase; TALA, transaldolase; TKT, transketolase; TPI, triose-phosphate isomerase; PGI, glucose 6-phosphate isomerase; RBPC, ribulose 1,5-bisphosphate carboxylase-oxygenase; PRUK, phosphoribulokinase; PFK, phosphofructokinase; EX_co2_e, exchange reaction of CO2; EX_no2_e, exchange reaction of NO2; ADK, adenylate kinase; GND, phosphogluconate dehydrogenase; G6PDH2r, glucose 6-phosphate dehydrogenase; PGL, 6-phosphogluconolactonase. (TIF) [file pcbi.1009828.s002.tif]

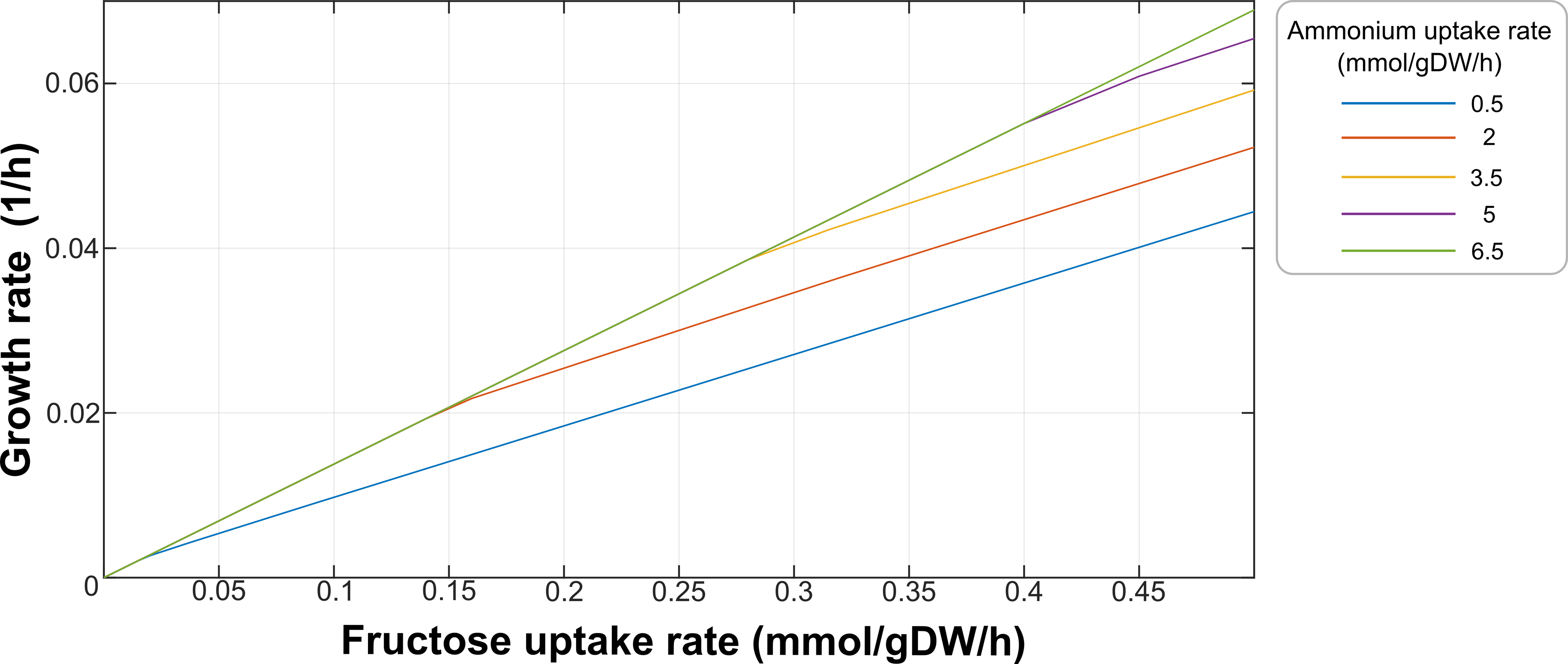

Supplement: S2 Fig — (TIF) [file pcbi.1009828.s003.tif]

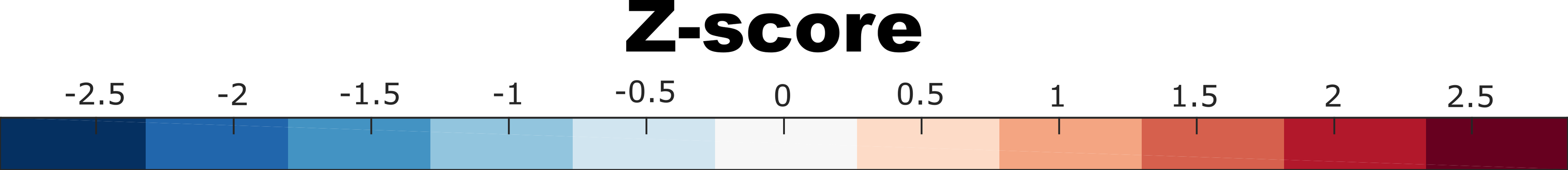

Supplement: S3 Fig — (TIF) [file pcbi.1009828.s004.tif]
